# Effects of high-flow nasal cannula and non-invasive ventilation on inspiratory effort in hypercapnic patients with chronic obstructive pulmonary disease: a preliminary study

**DOI:** 10.1186/s13613-019-0597-5

**Published:** 2019-10-22

**Authors:** Nuttapol Rittayamai, Prapinpa Phuangchoei, Jamsak Tscheikuna, Nattakarn Praphruetkit, Laurent Brochard

**Affiliations:** 1 Division of Respiratory Diseases and Tuberculosis, Department of Medicine, Faculty of Medicine Siriraj Hospital, Mahidol University, 2 Prannok Road, Bangkoknoi, Bangkok, 10700 Thailand; 2 Department of Emergency Medicine, Faculty of Medicine Siriraj Hospital, Mahidol University, Bangkok, Thailand; 3 Keenan Research Centre, Li Ka Shing Knowledge Institute, St. Michael’s Hospital, Toronto, ON Canada; 4 Interdepartmental Division of Critical Care Medicine, University of Toronto, Toronto, ON Canada

**Keywords:** Chronic obstructive pulmonary disease, Esophageal pressure, High-flow oxygen therapy, Non-invasive ventilation, Respiratory failure

## Abstract

**Background:**

Non-invasive ventilation (NIV) is preferred as the initial ventilatory support to treat acute hypercapnic respiratory failure in patients with chronic obstructive pulmonary disease (COPD). High-flow nasal cannula (HFNC) may be an alternative method; however, the effects of HFNC in hypercapnic COPD are not well known. This preliminary study aimed at assessing the physiologic effects of HFNC at different flow rates in hypercapnic COPD and to compare it with NIV.

**Methods:**

A prospective physiologic study enrolled 12 hypercapnic COPD patients who had initially required NIV, and were ventilated with HFNC at flow rates increasing from 10 to 50 L/min for 15 min in each step. The primary outcome was the effort to breathe estimated by a simplified esophageal pressure–time product (sPTP_es_). The other studied variables were respiratory rate, oxygen saturation (SpO_2_), and transcutaneous CO_2_ pressure (PtcCO_2_).

**Results:**

Before NIV initiation, the median [interquartile range] pH was 7.36 [7.28–7.37] with a PaCO_2_ of 51 [42–60] mmHg. sPTP_es_ per minute was significantly lower with HFNC at 30 L/min than 10 and 20 L/min (*p *< 0.001), and did not significantly differ with NIV (median inspiratory/expiratory positive airway pressure of 11 [10–12] and [5–5] cmH_2_O, respectively). At 50 L/min, sPTPes per minute increased compared to 30 L/min half of the patients. Respiratory rate was lower (*p *= 0.003) and SpO_2_ was higher (*p *= 0.028) with higher flows (30–50 L/min) compared to flow rate of 10 L/min and not different than with NIV. No significant differences in PtcCO_2_ between NIV and HFNC at different flow rates were observed (*p *= 0.335).

**Conclusions:**

Applying HFNC at 30 L/min for a short duration reduces inspiratory effort in comparison to 10 and 20 L/min, and resulted in similar effect than NIV delivered at modest levels of pressure support in hypercapnic COPD with mild to moderate exacerbation. Higher flow rates reduce respiratory rate but sometimes increase the effort to breathe. Using HFNC at 30 L/min in hypercapnic COPD patients should be further evaluated. *Trial registration* Thai Clinical Trials Registry, TCTR20160902001. Registered 31 August 2016, http://www.clinicaltrials.in.th/index.php?tp=regtrials&menu=trialsearch&smenu=fulltext&task=search&task2=view1&id=2008.

## Background

Exacerbation of chronic obstructive pulmonary disease (COPD) is defined as an acute worsening of respiratory symptoms, including increased dyspnea, cough, and sputum production, that results in the requirement for additional treatment and hospitalization [1]. Acute hypercapnic respiratory failure frequently occurs in patients with moderate to severe COPD exacerbation, and this condition generally necessitates an emergency room visit and hospital admission [2, 3]. Non-invasive ventilation (NIV) has been demonstrated to reduce the intubation rate and to improve survival in COPD patients who could require ventilatory support, and it is recommended to use it in hypercapnic COPD patients with respiratory acidosis [4–6]. However, skill of the caregivers is important to the success of this technique. In addition, patient tolerance is often poor due to patient discomfort and adverse effects frequently occur during its use, such as skin damage, air leaks, and claustrophobia [7–9].

High-flow nasal cannula (HFNC) is an oxygen device that delivers gas through a special large-bore nasal cannula. The current system can provide heated and humidified gas with a maximum flow rate of 60 L per minute (L/min) and an adjustable oxygen fraction (FiO_2_) from 21% to 100% [10]. The main mechanisms of HFNC include generation of a small amount of positive end-expiratory pressure (PEEP) varying from 1 to 7 cmH_2_O, wash out of nasopharyngeal dead space and provision of heat and humidity to reduce dryness symptom and facilitate secretion clearance [11]. Moreover, HFNC can improve oxygenation, modify breathing pattern, and decrease inspiratory effort. Several studies demonstrated physiological and clinical benefits of HFNC in patients with acute hypoxemic respiratory failure [12, 13] and for prevention of postextubation failure [14, 15]. The washing out of airway dead space by HFNC can increase CO_2_ clearance and improve alveolar ventilation, both of which could be beneficial in patients with hypercapnia. However, evidence supporting the efficacy of HFNC in hypercapnic respiratory failure is still limited. Furthermore, the maximum effect of HFNC for reducing inspiratory effort in acute hypoxemic respiratory failure occurs when using the highest flow rate, but we have had no data regarding the optimum flow rate of HFNC in patients with acute hypercapnic respiratory failure. Accordingly, the aim of this preliminary study was to investigate the physiologic effects of HFNC at different flow rates in patients with mild to moderate exacerbation, compared to the effect of NIV on inspiratory effort and other physiologic variables in COPD patients with hypercapnia.

## Methods

### Subjects and study design

This prospective physiologic study was conducted in the Respiratory Intensive Care Unit and the Respiratory Ward of the Division of Respiratory Diseases and Tuberculosis, Department of Medicine, Faculty of Medicine Siriraj Hospital, Mahidol University, Bangkok, Thailand during September 2016–May 2017. This study was approved by the Siriraj Institutional Review Board (SIRB) (COA no. 455/2559[EC4]), and was registered in the Thai Clinical Trials Registry (TCTR) (reg. no. 20160902001). Written informed consent to participate was obtained from each subject or their relatives.

We enrolled patients who had a known diagnosis of COPD with post-bronchodilator forced expiratory volume at 1 s/forced vital capacity (FEV_1_/FVC) < 70% [1], who were 40–85 years old and who had presented an exacerbation that initially required NIV based on at least two of the following criteria [16]:

- Respiratory rate > 24 breaths/min.
- Use of respiratory accessory muscles or paradoxical motion of the abdomen.
- Acute respiratory acidosis (arterial pH ≤ 7.35 and/or PaCO_2_ ≥ 45 mmHg).

After the initial management and stabilization with NIV, patients were considered for the study. Patients were not enrolled in the study if they had any of the following exclusion criteria: arterial pH < 7.25, hemodynamic instability, persistent hypoxemia despite supplemental oxygen therapy, diminished consciousness or uncooperative, active hemoptysis, pneumothorax, and/or contraindication for esophageal balloon catheter insertion, such as recent upper airway/esophageal surgery or active upper gastrointestinal bleeding.

### Device description

The HFNC device (Airvo-2^™^; Fisher & Paykel Healthcare, Auckland, New Zealand) consisted of a flow generator (up to 60 L/min), an air–oxygen blender that allows for adjustment of FiO_2_ from 21 to 100%, and an auto-fill MR 290 heated chamber. The gas mixture at 34–37 °C was delivered via a single-limb heated breathing tube to the patient via the Optiflow™ nasal cannula (Fisher & Paykel, Auckland, New Zealand). The dedicated NIV machine (BIPAP Vivo 40; Breas Medical AB, Mölnlycke, Sweden or Respironics V60; Philips Healthcare, Best, the Netherlands) was applied via an oronasal mask and was connected to an active humidification system (VH2000, VADI Medical Technology, Taoyuan, Taiwan). An appropriately sized oronasal mask was chosen to minimize leaks and to optimize patient comfort. The NIV settings, including inspiratory positive airway pressure, expiratory positive airway pressure, respiratory rate, and FiO_2_, were clinically adjusted by an attending physician and not modified during the study period.

### Study protocol

Subjects meeting all of the eligibility criteria and none of the exclusion criteria were enrolled. An esophageal balloon catheter (Cooper Surgical, Inc., Trumbull, Connecticut, USA) was inserted through the nose and positioned in the lower one-third of the esophagus. The balloon was filled with 0.5 mL of air and connected to a pressure transducer (BIOPAC Systems, Goleta, California, USA). To confirm the position of the esophageal catheter, the presence of cardiac oscillations was checked and gentle pressure on the abdomen was applied to verify the absence of gastric pressure fluctuations. Esophageal pressure (P_es_) was recorded using an MP150 Data Acquisition System and AcqKnowledge Data Acquisition and Analysis Software (both BIOPAC Systems, Goleta, California, USA).

**Fig. 1:**
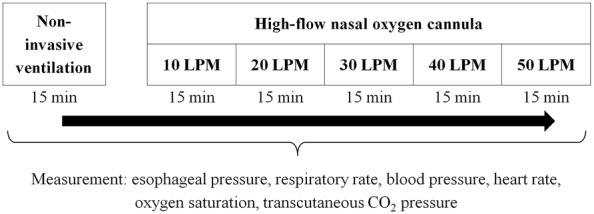
Study protocol

At inclusion, subjects were ventilated with NIV using their clinical settings for 15 min, after which they were switched to HFNC starting at a flow rate of 10, with subsequent progressive increases to 20, 30, 40, and 50 L/min. The NIV step and all 5 HFNC steps were applied for 15 min each including a recording period of 5 min at the end (Fig. 1). During HFNC, the FiO_2_ was adjusted to achieve oxygen saturation measured by pulse oximetry (SpO_2_) of at least 92%, and then this adjustment was kept constant until the end of the study protocol. To enhance the maximum effect of HFNC, we encouraged patients to breathe with their mouth closed as often as possible. After completing the study, the type and settings of respiratory support were decided by the attending physician.

### Data collection

Baseline demographic and clinical data, included age, gender, body mass index, co-morbidity, and most recent pulmonary function test were collected. Acute Physiologic and Chronic Health Evaluation (APACHE) II score and arterial blood gas were evaluated at the time of enrollment during NIV session. SpO_2_ and transcutaneous CO_2_ pressure (PtcCO_2_) were continuously recorded throughout the study using a SenTec Digital Monitoring System (SenTec, Therwil, Switzerland). Other physiologic variables, including respiratory rate, blood pressure, and heart rate, were recorded immediately after starting the protocol and at the end of each step.

From *P*_es_, we used the recorded waveforms during the last 2 min of each step to calculate *P*_es_ swing and esophageal pressure–time product (PTP_es_). The average value of *P*_es_ swing (cmH_2_O), PTP_es_ per minute (cmH_2_O × s × min^−1^), and PTP_es_ per breath (cmH_2_O × s) as an index of inspiratory effort were calculated using a dedicated software program (Sistema Respiratorio, Barcelona, Spain). Since measurement of chest wall elastance in non-intubated patients is not possible, and determining the phase of inspiration without airflow signal is also very difficult, we modified the calculation of PTP_es_ per breath by integrating the area under the *P*_es_ signal from the onset of negative deflection to the return of *P*_es_ to baseline. This technique was used and reported in a previous study [17]; we refer to it as a “simplified PTP_es_” (sPTP_es_). sPTP_es_ per minute was obtained by multiplying sPTP_es_ per breath by respiratory rate.

### Outcomes

The primary outcome was inspiratory effort as evaluated by sPTP_es_ per minute during NIV and during HFNC at different flow rates. Other physiologic variables included sPTP_es_ per breath, *P*_es_ swing, respiratory rate, SpO_2_, PtcCO_2_, blood pressure, and heart rate.

### Statistical analysis

There was no previous study evaluating patient inspiratory effort during HFNC in COPD patients with acute exacerbation. We performed a pilot exploratory study by enrolling 12 patients in this study. Normality of data distribution was assessed by the Shapiro–Wilk test. Normally distributed variables are expressed as mean ± standard deviation, and were analyzed by repeated measures analysis of variance (ANOVA) followed by a post hoc pairwise comparison with Bonferroni adjustment. Non-normally distributed variables are expressed as median and interquartile range, and were compared by Friedman’s two-way ANOVA by ranks with a Dunn’s test post hoc pairwise comparison with Bonferroni correction. Categorical variables are expressed as frequency and percentage. Data were analyzed using PASW Statistics version 18 (SPSS, Inc., Chicago, Illinois, USA). A two-sided *p *< 0.05 was considered as statistically significant.

## Results

Twelve patients were included, with a mean age of 74 ± 11 years. Post-bronchodilator FEV_1_ and FEV_1_/FVC were 34% [IQR 31–47] of predicted and 45% [IQR 35–55], respectively. Patients were enrolled a median of 17 h [IQR 5–68] after initiation of NIV. Other baseline characteristics are shown in Table 1.

**Table 1:** Baseline patient demographic and clinical characteristics (*n* = 12)

Variables	*N* = 12
Age, years	74.0 ± 11.0
Male gender, *n* (%)	9 (75%)
Body mass index, kg/m^2^	22 ± 3
Long-term oxygen therapy, *n* (%)	3 (25%)
Comorbidity, *n* (%)	
Diabetes mellitus	2 (17%)
Hypertension	8 (67%)
Coronary artery disease	3 (25%)
Chronic kidney disease	4 (33%)
Others	2 (17%)
Post-bronchodilator FEV_1_/FVC, %	45 [35–55]
Post-bronchodilator FEV_1_, % predicted	34 [31–47]
APACHE II at enrollment	12 ± 4
Arterial blood gas at enrollment	
pH	7.36 [7.28–7.37]
PaCO_2_, mmHg	51 [42–60]
PaO_2_, mmHg	139 [80–233]
Time from initiation of NIV to enrollment, hours	17 [5–68]
Non-invasive ventilation setting at enrollment	
Inspiratory positive airway pressure, cmH_2_O	11 [10–12]
Expiratory positive airway pressure, cmH_2_O	5 [5]
Tidal volume, mL	425 [370–514]
Oxygen flow rate, liters per minute	4 [3–5]

Continuous variables were presented as mean ± SD or median [interquartile range] and categorical variables were presented as absolute value (%)

*APACHE* acute physiologic and chronic health evaluation, *FEV*_1_ forced expiratory volume at 1 s, *FVC* forced vital capacity, *PaCO*_2_ arterial partial pressure of carbon dioxide, *PaO*_2_ arterial partial pressure of oxygen

### Inspiratory effort and respiratory rate during HFNC and NIV

**Fig. 2:**
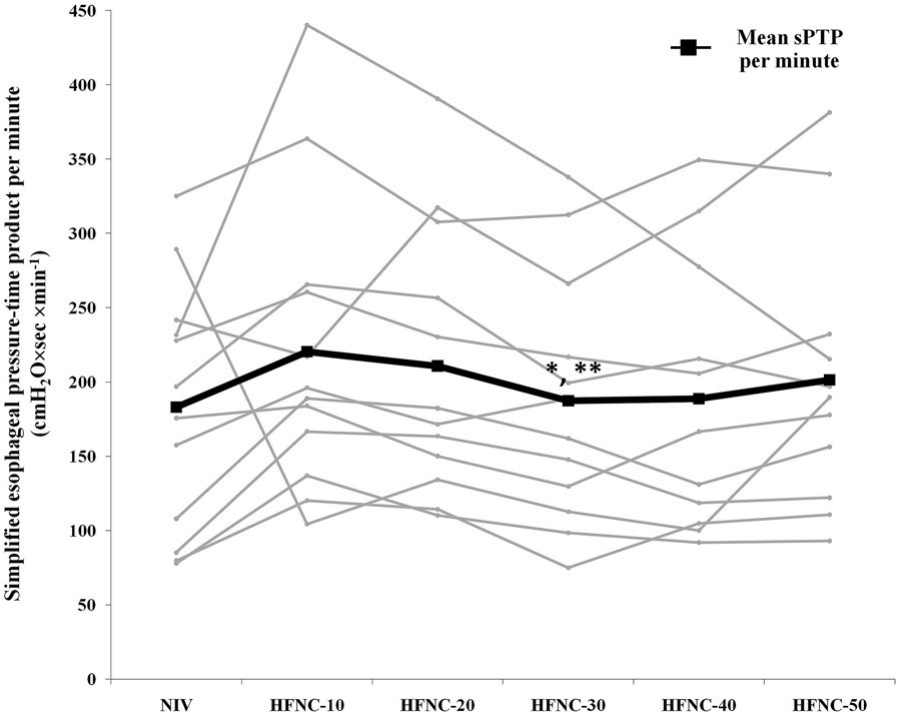
Individual data and mean value of simplified esophageal pressure–time product (sPTP_es_) per minute during non-invasive ventilation (NIV) and high-flow nasal cannula (HFNC) at different flow rates. *Indicates *p *< 0.05 in comparison to HFNC at a flow rate of 10 L/min, **Indicates *p *< 0.05 in comparison to HFNC at a flow rate of 20 L/min

Changes in sPTP_es_ per breath and per minute between HFNC at different flow rates and NIV are shown in Table 2. Changes in sPTP_es_ per breath were relatively modest and non-significant. sPTP_es_ per minute was significantly lower with HFNC at a flow rate of 30 L/min compared to flow rates of 10 L/min and 20 L/min (187 ± 84, 220 ± 100 cmH_2_O × s × min^−1^ and 211 ± 90 cmH_2_O × s × min^−1^, respectively; *p *< 0.01). sPTP_es_ per minute was not different between HFNC at a flow rate of 30 L/min and NIV (187 ± 84 vs. 183 ± 84 cmH_2_O × s × min^−1^; *p *= 0.839). In half of the subjects, sPTP_es_ per minute increased progressively when increasing the flow rate of HFNC to 50 L/min (Fig. 2); however, the overall increase was not statistically significant (30 L/min vs. 50 L/min = 187 ± 84 vs. 201 ± 86 cmH_2_O × s×min^−1^; *p *= 0.269).

**Table 2:** Simplified esophageal pressure–time product, respiratory variables, and hemodynamic variables during non-invasive ventilation and high-flow nasal cannula at different flow rates

Variables	NIV	HFNC-10	HFNC-20	HFNC-30	HFNC-40	HFNC-50	*p*
sPTP_es_ per breath (cmH_2_O × s)	9 ± 4	11 ± 5	11 ± 6	10 ± 6	10 ± 5	10 ± 4	0.090
sPTP_es_ per minute (cmH_2_O × s × min^−1^)	183 ± 84	220 ± 100	211 ± 90	187 ± 84*,**	189 ± 87	201 ± 86	< *0.001*
RR (breaths/min)	25 [20–28]	24 [20–29]	23 [18–28]	21 [18–27]*	21 [18–27]^*^	21 [18–26]^*^	*0.003*
SpO_2_ (%)	96 ± 2	95 ± 3	96 ± 3*	97 ± 3*,**	97 ± 2*,**	98 ± 2*,**, ^γ^	*0.028*
PtcCO_2_ (mmHg)	42 ± 7	41 ± 7	41 ± 8	41 ± 7	41 ± 7	41 ± 7	0.335
MAP (mmHg)	99 ± 22	94 ± 15	92 ± 9	91 ± 12	89 ± 12	91 ± 14	0.179
HR (beats/min)	96 ± 16	97 ± 15	94 ± 13	93 ± 14	94 ± 16	95 ± 17	0.174
Oxygen flow (L/min) or Set FiO_2_	4 [3–5]	0.35 [0.30–0.40]	0.35 [0.30–0.40]	0.35 [0.30–0.40]	0.35 [0.30–0.40]	0.35 [0.30–0.40]	–

Continuous variables were presented as mean ± SD or median [interquartile range] and categorical variables were presented as absolute value (%)

*HFNC* high-flow nasal cannula,* HR* heart rate,* MAP* mean arterial pressure,* NIV* non-invasive ventilation,* sPTP*_es_, simplified esophageal pressure–time product,* PtcCO*_2_, transcutaneous carbon dioxide pressure,* RR* respiratory rate,* SpO*_2_, oxygen saturation

* Indicates *p *< 0.05 compared with HNFC-10, ** Indicates *p *< 0.05 compared with HFNC-20, ^γ^Indicates *p *< 0.05 compared with NIV

**Fig. 3:**
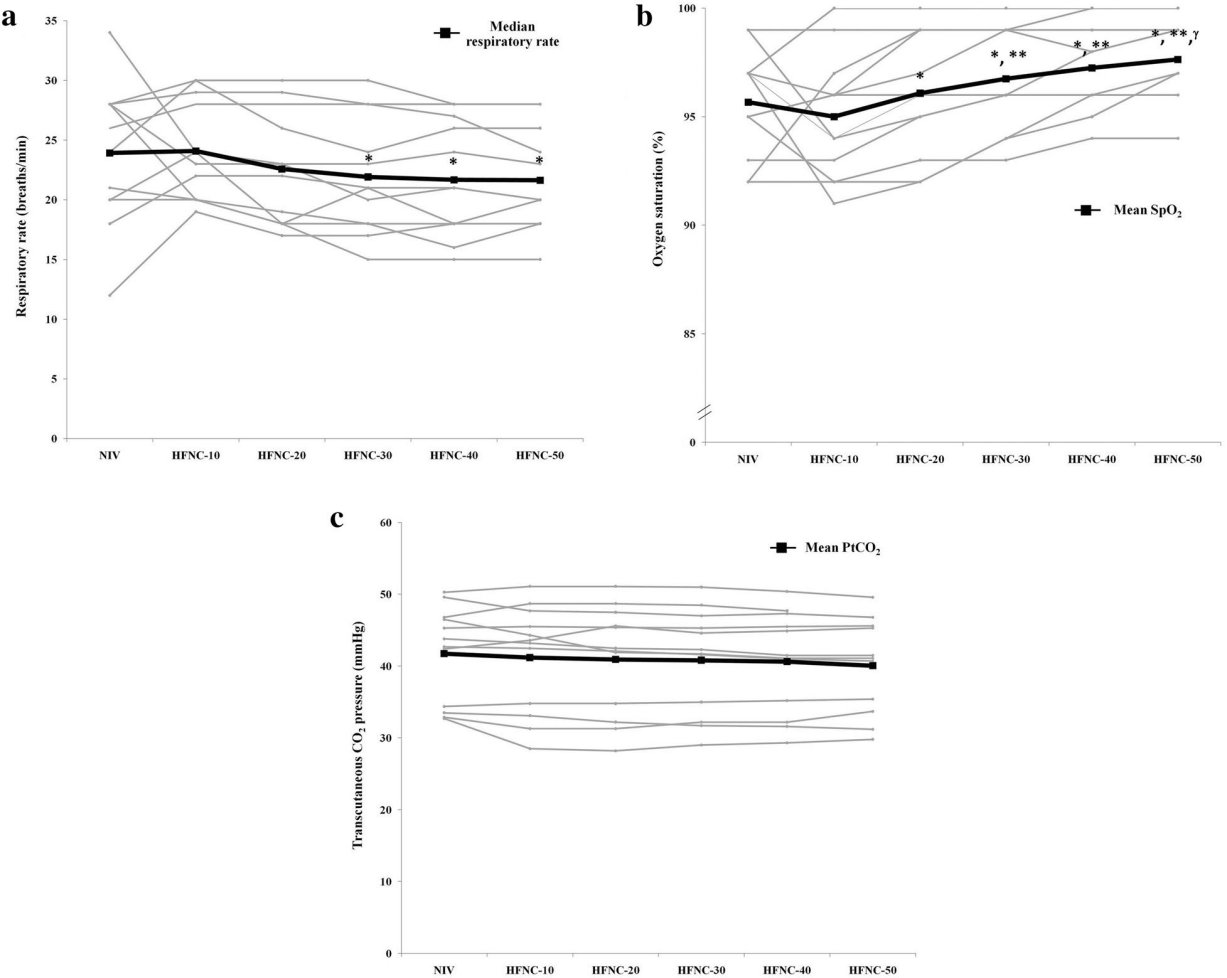
Individual data of **a** respiratory rate, **b** oxygen saturation (SpO_2_), and **c** transcutaneous CO_2_ pressure (PtcCO_2_) during non-invasive ventilation (NIV) and high-flow nasal cannula (HFNC) at different flow rates. *Indicates *p *< 0.05 compared with HFNC at a flow rate of 10 L/min, **Indicates *p *< 0.05 compared with HFNC at a flow rate of 20 L/min, ^γ^Indicates *p *< 0.05 compared with NIV

HFNC at flow rate of 30, 40, and 50 L/min, respiratory rate was significantly lower compared to HFNC at a flow rate of 10 L/min (*p *= 0.003). There was no significant difference in respiratory rate between HFNC at flow rates of 30–50 L/min and NIV (Fig. 3 and Table 2).

### Other physiological variables

With constant FiO_2_, HFNC at a higher flow rate resulted in significantly increased SpO_2_ compared to that of HFNC at a flow rate of 10 L/min (Table 2). Furthermore, SpO_2_ was significantly higher in HFNC at a flow rate of 50 L/min compared to NIV (98 ± 2% vs. 96 ± 2%; *p *= 0.024). No significant difference in PtcCO_2_ between HFNC at any flow rate and NIV was found (*p *= 0.335). The individual data of SpO_2_ and PtcCO_2_ are shown in Fig. 3.

No significant difference in mean arterial pressure or heart rate was observed between NIV and HFNC at any flow rate (Table 2).

### Adverse events

No adverse events were observed during either HFNC or NIV in this study. All patients tolerated both study interventions until the end of the study.

## Discussion

In this preliminary, short-term physiological study, we evaluated the effects of HFNC at different flow rates and compared to NIV in patients with mild to moderate COPD exacerbation who had initially been managed and stabilized with NIV. The primary outcome demonstrated that HFNC at a flow rate of 30 L/min significantly reduced inspiratory effort as assessed by sPTP_es_ per minute compared to HFNC at a flow rate of 10 L/min; at this flow rate, HFNC was comparable to NIV for reducing sPTP_es_ per minute. Although we did not observe a significant difference in sPTP_es_ per breath between HFNC at flow rates of 30 and 10 L/min, decreased sPTP_es_ per minute when increasing the flow rate may be explained by a significant reduction in respiratory rate. Interestingly, we observed a trend of increasing sPTP_es_ per minute, when increasing the HFNC flow rate from 30 to 50 L/min at least in some patients. The effect of increasing the flow rate of HFNC on inspiratory effort in our study was different from the effect observed and reported in previous studies. Two studies in patients with acute hypoxemic respiratory failure by Mauri, et al. found that PTP_es_ per minute progressively decreased [18], and that patients were more comfortable [19] when the HFNC flow rate was uptitrated from 30 to 60 L/min. A study by Delorme and colleagues [20] in 12 patients recovering from acute respiratory failure also reported significant decrease in PTP_es_ per minute when increasing the HFNC flow rate from 20 to 60 L/min. However, they did not find any significant difference in PTP_es_ per minute in the subgroup of patients with hypercapnic respiratory failure. We have no clear explanation why an increase in the HFNC flow rate from 30 to 50 L/min in our study led to increased inspiratory effort in half of COPD patients with exacerbation; however, patient discomfort, worsening dynamic hyperinflation or increased resistance to breathe could explain an observed increased inspiratory effort with HFNC at a flow rate of 50 L/min. A study comparing HFNC at 30 L/min with conventional oxygen therapy in patients with stable COPD found significant increases in end-expiratory lung volume with HFNC [21]. The increase in end-expiratory lung volume with HFNC may have aggravated dynamic hyperinflation and effort to breathe in some COPD patients in our study.

Several studies have demonstrated that HFNC improved work of breathing and breathing pattern in patients with acute hypoxemic respiratory failure when compared to conventional oxygen therapy [22], and it was not found to be inferior to NIV [17]; however, no previous study has investigated the effects of HFNC on inspiratory effort in COPD patients with exacerbation. Somewhat similar to our study, Sklar and colleagues [23] conducted a study that compared HFNC and NIV in 15 patients with cystic fibrosis who developed acute hypercapnia. They found that HFNC was not inferior to NIV with respect to diaphragmatic work as assessed by diaphragm thickening fraction measured by ultrasound. Other short-term physiological studies of HFNC in patients with stable COPD found that HFNC significantly reduced respiratory rate and PtcCO_2_ in comparison to low-flow oxygen therapy [21, 24]. In contrast to our study, Pisani et al. [25] demonstrated that diaphragm pressure–time product was significantly lower with NIV than with HFNC at flow rate of 20 and 30 L/min in patients with stable COPD. This difference, however, may be explained by longer duration of the intervention and different NIV settings. Several mechanisms of HFNC that influence reduced inspiratory effort have been proposed, including higher flow rate of gas matching the patient’s demand [10, 26], washing out anatomic dead space that results in reduction of ineffective ventilation [27–29], and the effect of external PEEP to overcome intrinsic PEEP caused by dynamic airway collapse in COPD with acute exacerbation [30].

In this study, higher flow rate of HFNC improved oxygenation compared to NIV. However, we did not find a significant reduction in PtcCO_2_ with HFNC. Reduction of anatomic dead space and subsequent CO_2_ washout is the mechanism that has been proposed to explain the decrease in PtcCO_2_ [28]. A study in 30 patients with stable COPD who had an indication for long-term oxygen therapy by Fraser et al. [21] found that HFNC at a flow rate of 30 LPM significantly improved oxygenation and reduced PtcCO_2_ when compared to conventional oxygen therapy. A study by Braunlich et al. [31] compared HFNC, nasal CPAP, and nasal NIV in 67 hospitalized patients with COPD and found that increasing the flow rate of HFNC from 20 to 30 L/min enhanced CO_2_ clearance and lowered PaCO_2_. They did not find any difference between HFNC at a flow rate of 30 LPM and NIV in terms of reduction in PaCO_2_. Conversely, a randomized crossover study in 24 hospitalized patients with COPD exacerbation by Pilcher and colleagues [32] did not find significant difference in PtcCO_2_ when compared between HFNC at a flow rate of 35 L/min and standard nasal prongs. A study by Atwood et al. compared HFNC at a flow rate of 35 L/min with low-flow oxygen therapy in 32 stable COPD patients. They found that HFNC markedly reduced respiratory rate with no significant changes in tidal volume or PaCO_2_, which suggested more efficient ventilation with HFNC. Thus, the lack of significant change in PtcCO_2_ in our study might be explained by the reduction in respiratory rate, and we assume that alveolar ventilation was improved when increasing the flow rate of HFNC.

### Limitations

This study has some limitations. First, this preliminary study had a small number of enrolled subjects. Second, the study interventions were not randomized then an effect of the treatment duration on many physiologic variables may not be ruled out. Third, we did not measure delivered FiO_2_ during NIV, tidal volume during HFNC, and level of intrinsic PEEP due to device-related limitations. Forth, the time spent on each step was relatively short and may not have been long enough to detect significant differences in physiologic effects between HFNC and NIV, in particular change in PtcCO_2_. Fifth, the level of pressure support during NIV in our study was low compared to the levels reported in previous studies in COPD patients with exacerbation, and most patients were stabilized before enrollment in the study. This factor may influence a bias toward HFNC in terms of lowering inspiratory effort. Last, the level of patient discomfort was not assessed in our study, and this factor could limit the efficacy of these techniques. Thus, another physiological study with longer duration of the treatment and also a larger randomized controlled study of HFNC in COPD patients with acute hypercapnic respiratory failure are needed to confirm our results and to further elucidate the efficacy of HFNC in this patient population.

## Conclusions

After a short duration of HFNC at 30 L/min, inspiratory effort as determined by sPTP_es_ per minute decreases similar to the reduction effectuated by NIV delivered at modest levels of pressure support in hypercapnic COPD patients with mild to moderate exacerbation in comparison to HFNC at 10 and 20 L/min. Higher flow rates reduce respiratory rate and improves oxygenation but sometimes increase the effort to breathe. However, higher HFNC flow rate do not provide significant change in PaCO_2_. Our results suggest that HFNC at 30 L/min might be optimal in many hypercapnic COPD patients with mild to moderate exacerbation and should be tested in the future study.

## Data Availability

The datasets supporting the conclusions of this article are included within the article.

## Abbreviations

APACHE: Acute Physiologic and Chronic Health Evaluation
COPD: chronic obstructive pulmonary disease
FEV_1_: forced expiratory volume at 1 s
FiO_2_: oxygen fraction
FVC: forced vital capacity
HFNC: high-flow nasal cannula
LPM: liters per minute
NIV: non-invasive ventilation
PaCO_2_: arterial partial pressure of carbon dioxide
PaO_2_: arterial partial pressure of oxygen
PEEP: positive end-expiratory pressure
P_es_: esophageal pressure
PtcCO_2_: transcutaneous carbon dioxide pressure
sPTP_es_: simplified esophageal pressure–time product
SpO_2_: oxygen saturation by pulse oximetry
